# Impact of androgen deprivation therapy on the thymus and the production of naïve T-cells

**DOI:** 10.1186/2051-1426-1-S1-P83

**Published:** 2013-11-07

**Authors:** Ravi A Madan, Hakim Frances, Caroline Jochems, Christopher R Heery, Geraldine C O’Sullivan, Harpreet Singh, Ira Surolia, Kwong Y Tsang, Ronald Gress, Jeffery Schlom, James L Gulley

**Affiliations:** 1 Laboratory of Tumor Immunology & Biology, NCI, Bethesda, MD, USA; 2 Medical Oncology Branch, NCI, Bethesda, MD, USA; 3 Experimental Transplantation & Immunology, NCI, Bethesda, MD, USA

## Purpose

A clinical trial was conducted employing androgen deprivation therapy (ADT) +/- vaccine in patients (pts) with rising prostate specific antigen (PSA) after definitive therapy but no disease visible on bone or computed tomography (CT) scan. The primary endpoint was to determine if a whole tumor cell vaccine could prolong PSA recovery, but a key secondary endpoint was to evaluate the impact of ADT on the thymus and production of naïve T-cells. Previous studies suggest that ADT regenerates the thymus (in mice) and increases naïve T-cell production in humans [1].

## Methods

All pts were treated with a 3 month dose of goserelin (ADT) as part of an intermittent therapy approach, consistent with the standard of care for this population. Once a PSA decline was confirmed at 12 weeks, pts were randomized to treatment with vaccine or placebo administered via 2 intradermal injections each at 4 sites in the torso. The vaccine consisted of 3 irradiated prostate cancer cell lines. Pts received injections at weeks 1, 3, 5 and then monthly to complete 12 months of therapy. Pts had follow-up evaluations of testosterone (T) levels, thymic measurements on CT scan and assessments of naïve T-cells. Thymic evaluations were based on a previously established radiographic measure, the thymic index [2]. Changes in naïve CD 4 T-cells, defined as CD45RA+CD31+, and T-cell receptor excision circles (TRECs) were also evaluated. Wilcoxon matched pairs signed rank analyses was used in this analysis.

## Results

33 pts were evaluable after response to ADT. The median age, PSA and T were 62 years, 2.2 ng/ml and 361 ng/dl, respectively. Median time to T recovery after a single 3-month shot of the GnRH agonist was 7 months (mos), range: 3-15 mos. Six mos after ADT, only 2 pts had more than minimal changes in thymus size (thymic index >2) on CT, however significant changes in naïve T-cell populations were detected in 20 evaluable pts. Naïve CD4 cells increased from a median of 16.4% of CD4+ T-cells to 20.5%(p=0.0014) and TRECs increased from 93/100,000 cells to 147/100,000 cells (p=0.0025).

## Conclusion

These data suggest that ADT could have a significant impact on the production of naïve T-cells despite minimal impact on the actual size of the thymus at 6 months. The ultimate clinical impact of a change in immune parameters may not be immediately clear given the short duration of follow-up in this trial. These data further support the hypothesis that immune stimulating therapies could be combined with ADT to enhance immune responses.
